# Takotsubo Syndrome: Translational Implications and Pathomechanisms

**DOI:** 10.3390/ijms23041951

**Published:** 2022-02-10

**Authors:** Xuehui Fan, Guoqiang Yang, Jacqueline Kowitz, Ibrahim Akin, Xiaobo Zhou, Ibrahim El-Battrawy

**Affiliations:** 1 First Department of Medicine, Medical Faculty Mannheim, University Medical Centre Mannheim (UMM), University of Heidelberg, 68167 Mannheim, Germany; Xuehui.Fan@medma.uni-heidelberg.de (X.F.); jm.k@live.com (J.K.); Ibrahim.Akin@umm.de (I.A.); 2 Key Laboratory of Medical Electrophysiology, Ministry of Education and Medical Electrophysiological Key Laboratory of Sichuan Province, Collaborative Innovation Center for Prevention of Cardiovascular Diseases, Institute of Cardiovascular Research, Southwest Medical University, Luzhou 646000, China; 3 DZHK (German Center for Cardiovascular Research), Partner Site, Heidelberg-Mannheim, 68167 Mannheim, Germany; 4 Department of Acupuncture and Rehabilitation, the Affiliated Traditional Chinese Medicine Hospital of Southwest Medical University, Luzhou 646000, China; Guoqiangy.16@gmail.com; 5 Research Unit of Molecular Imaging Probes, Department of Radiologic Technology, Faculty of Associated Medical Sciences, Chiang Mai University, Chiang Mai 50200, Thailand

**Keywords:** Takotsubo syndrome, pathophysiological mechanism, human-induced pluripotent stem cell-derived cardiomyocytes, catecholamines, precision medicine

## Abstract

Takotsubo syndrome (TTS) is identified as an acute severe ventricular systolic dysfunction, which is usually characterized by reversible and transient akinesia of walls of the ventricle in the absence of a significant obstructive coronary artery disease (CAD). Patients present with chest pain, ST-segment elevation or ischemia signs on ECG and increased troponin, similar to myocardial infarction. Currently, the known mechanisms associated with the development of TTS include elevated levels of circulating plasma catecholamines and their metabolites, coronary microvascular dysfunction, sympathetic hyperexcitability, inflammation, estrogen deficiency, spasm of the epicardial coronary vessels, genetic predisposition and thyroidal dysfunction. However, the real etiologic link remains unclear and seems to be multifactorial. Currently, the elusive pathogenesis of TTS and the lack of optimal treatment leads to the necessity of the application of experimental models or platforms for studying TTS. Excessive catecholamines can cause weakened ventricular wall motion at the apex and increased basal motion due to the apicobasal adrenoceptor gradient. The use of beta-blockers does not seem to impact the outcome of TTS patients, suggesting that signaling other than the beta-adrenoceptor-associated pathway is also involved and that the pathogenesis may be more complex than it was expected. Herein, we review the pathophysiological mechanisms related to TTS; preclinical TTS models and platforms such as animal models, human-induced pluripotent stem cell-derived cardiomyocyte (hiPSC-CM) models and their usefulness for TTS studies, including exploring and improving the understanding of the pathomechanism of the disease. This might be helpful to provide novel insights on the exact pathophysiological mechanisms and may offer more information for experimental and clinical research on TTS.

## 1. Introduction

Takotsubo syndrome (TTS), also known as Takotsubo cardiomyopathy (TTC) or broken heart syndrome or stress-induced cardiomyopathy, is a type of acute heart failure syndrome characterized by an acute, transient and reversible left ventricular (LV) systolic dysfunction with apical ballooning and ST-segment elevation or T-wave inversion in the absence of obstructive coronary artery disease (CAD), which is often associated with emotional or physical stress [1,2]. TTS has been reported in adults of all ages and even in children [3]. TTS was first described in Japan in 1990, in five cases of typical cardiac arrest patients suffering from chest pain with abnormal electrocardiogram, similar to acute myocardial infarction (AMI), but without evidence of coronary artery stenosis on angiography (CAG) [4]. Importantly, the major criteria to differentiate TTS from AMI are the absence of significant obstructive CAD contributing to the extent of contraction abnormalities [5,6], and the recovery of the wall motion abnormality and ejection fraction abnormality. TTS and neurogenic stunning (NS) share many common features, although there are differences in the clinical and laboratory features between them [7,8]. For example, apical hypokinesia is more common in Takotsubo, whereas basal hypokinesia is typical of patients with NS [9]. The development of neurogenic cardiomyopathy (NC) is mainly associated with an elevated plasma norepinephrine (NE) level, which is mediated by neuronal NE rather than adrenal epinephrine (EPI) [10]. Both a surge in catecholamine secretion in the first hour of brain death and a deficiency in the following hours are causes of cardiac stunning and altered vascular reactivity in NS [11].

The suggested mechanisms associated with the development of TTS include elevated levels of circulating plasma catecholamines and its metabolites, coronary microvascular dysfunction, sympathetic hyperexcitability, inflammation, estrogen deficiency, basal hypercontractility with left ventricular outflow tract obstruction and signal trafficking/biased agonism, spasm of the epicardial coronary vessels, genetic predisposition and thyroidal dysfunction [12,13,14,15,16,17]. Since the pathogenesis of TTS contains probably a combination of multiple factors, the precise pathogenesis of TTS is difficult to fully explore. Contemporary analyses show that 20–25% of TTS patients have received beta-blocker drugs during their initial event and 40% during a second episode. However, no drug treatment has been found to be effective to prevent the recurrence or improve the outcome of TTS [18]. The lack of optimal treatment for TTS leads to the necessity of the application of experimental models or platforms for studying TTS. Several models, mainly focusing on animal models and human-induced pluripotent stem cell-derived cardiomyocyte (hiPSC-CM) models, have been used to simulate the disease and investigate possible pathogenic mechanisms. Each model has its own advantages and disadvantages. Herein, we focus on reviewing the existing TTS models and pathogenesis of TTS to gain a better understanding of the underlying mechanism of this disease. 

## 2. Clinical Characteristics and Epidemiology

Due to the initial unawareness of the disease and reversible character, the prevalence of TTS was underestimated. However, TTS has now become more and more widely recognized in almost all countries. Patients with TTS in South-East Asian are characterized by 82% being female patients, 89% with an apical variant, a high incidence of physical triggers and low cardiovascular mortality [19]. It has also been described in the United States and Europe, showing that patients with TTS have a higher prevalence of neurological or psychiatric diseases [20]. Studies on TTS have reported that the female sex predominated, mainly aged 56-84 years [21,22,23]. However, there is no huge difference in the clinical characteristics and the course of the disease between men and women, although physical stress is more likely to cause TTS with much worse outcomes in males compared to females [24,25,26]. Interestingly, given the increased awareness of the disease entity nowadays, male TTS patients are also growingly recognized with decreasing age, the highest in younger patients [20,27,28,29]. Approximately 10% of patients with TTS are younger than 50 years [29]. Women account for 90% of the cases, with an average age of approximately 65–75 years in most series, and women over 55 years of age have a five-fold increased risk of TTS [30].

TTS may be associated with severe and fatal complications. The potential triggers, including the emotional stress, inflammatory state, physical stress of cancer surgery, systemic antineoplastic therapy and radiation treatment, place patients with cancer at an elevated risk for developing TTS, with an 11% incidence rate of TTS being described over a 6-year follow-up among 275 patients with cancer [31,32]. Of 1750 TTS patients in a separate study, 290 (16.6%) had cancer, approximately equivalent to those with chronic obstructive pulmonary disease (16.2%) and CAD (15.3%) and much less than those with neurologic and psychiatric disorders (46.8%) [33]. Cardiovascular risk factors such as CAD, hypertension, diabetes and morbid obesity are less prevalent in TTS patients with cancer; however, these patients have a significantly higher all-cause mortality [34]. TTS patients with cancer experience atrial fibrillation more frequently than those without cancer. At our institution, 14% of patients with TTS had a malignancy already diagnosed at the time of TTS event, and further, 11% of patients were diagnosed with cancer during a 5-year follow-up [35,36]. TTS patients with adverse rhythm disorders have a lower survival rate compared to patients without them [37]. Left ventricular outflow tract obstruction (LVOTO) has been considered generally as a complication in the acute phase and after the recovery phase of TTS, since LVOTO that can add to the increase in LV pressure is associated with hemodynamic instability, mitral regurgitation, systolic anterior motion (SAM) and cardiogenic shock [38,39]. TTS shows hypercontractility of the basal and mid segments with apical wall dyskinesia [17]. However, the effect of LVOTO on the long-term prognosis of TTS is still poorly understood and deserves more attention.

Although myocarditis and Takotsubo cardiomyopathy may share a common mechanism, in the latter, β-adrenergic cardiotoxicity seems to be widespread [40]. Of note, not all myocarditis is TTS. It is crucial to distinguish myocarditis from TTS, and both cardiac magnetic resonance (CMR) and endomyocardial biopsy (EMB) were conducted to clarify diagnoses [41,42,43]. Late gadolinium enhancement (LGE) in myocarditis has a patchy distribution, whereas LGE is not usually present in TTS [44]. 

Currently, considerable progress has been made regarding the diagnosis, clinical characterization and first-line therapy of TTS, but the prognosis does not seem to be as benign as we thought, with a rate of death from any cause of 5.6% per patient/year and, also, with a significant percentage of in-hospital complications comparable to ACS [20]. The 30-day mortality rate of TTS patients is between ST-segment elevation myocardial infarction (STEMI) and non-STEMI (NSTEMI) [45].

## 3. Pathophysiology

**Figure 1:**
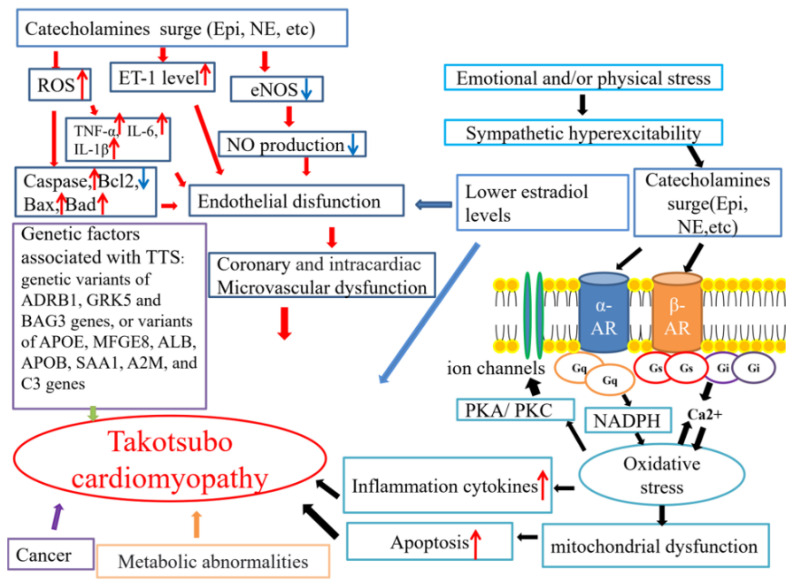
The possible mechanisms related to TTS. AR: adrenergic receptor; NE: norepinephrine; Epi: epinephrine. The red arrows mean increase, and the blue ones indicate decrease.

Until now, the precise pathophysiological mechanisms of TTS have remained controversial. Several mechanisms underlying TTS pathophysiology have been hypothesized (Figure 1).

### 3.1. A Surge in Catecholamines Caused by Sympathetic Excess

#### 3.1.1. Central Nervous System and Peripheral Nervous System

At present, it is believed that increasing sympathetic nervous system (SNS) activity, which results from functional and structural changes in the autonomic nervous system, plays a central part in the TTS pathogenesis [23,46,47]. The crosstalk between the brain and heart is critical to the development of TTS [20,48,49,50,51,52]. By a cross-sectional study, Hiestand et al. identified that TTS patients have significant anatomical differences compared with healthy control subjects in the limbic network, including the insula, amygdala, cingulate cortex and hippocampus, all of which are related to emotional processing, cognitive and autonomic nervous system control [50]. Among them, the resting metabolic activity of the amygdala, which is related to increased hemopoietic activity and increased arterial inflammation, significantly predicted the development of cardiovascular disease. Besides, amygdala activity was associated with perceived stress, which suggests that the amygdala may be a key structure linking stress to cardiovascular events [53]. Stress can lead to an increase in circulating catecholamines, glucocorticoids and inflammatory cytokines by promoting the activation of the sympathetic nervous system and the hypothalamic–pituitary–adrenal axis and can also increase the heart rate and blood pressure through the autonomic nervous system [53,54,55,56,57]. In long-standing cases of TTS, compared with the control group, the central autonomic neural network and the default mode network of TTS patients also had fewer functional connections at the whole-brain level [47,52]. The underlying pathophysiological mechanism of TTS may be related to the interaction between the brain and the heart.

#### 3.1.2. Emotional Stress and Physical Injury

Emotional stress or physical injury can cause sympathetic hyperexcitability which leads to the massive release of catecholamines, the pathophysiological basis of TTS. Intravenous administration of catecholamines and beta-agonists can cause the clinical features of TTS [58,59]. Kido et al. described that 68.2% of cases in 157 drug-induced TTS patients were related to catecholamine, while 14 cases appeared to be associated with chemotherapy-induced coronary vasospasm [60]. It has been demonstrated that the catecholamine levels were markedly higher among TTS patients than among those with Killip class III MI [61]. The increased response of the apical myocardium to sympathetic nerve stimulation may make the apex more vulnerable to sudden increases in the circulating catecholamine levels [62]. After a challenge with catecholamines leading to high blood pressure, basal akinesia was observed, while the apical area preserved function [63]. However, the high afterload made the apical areas shorten earlier and basal regions experience higher wall stress [63]. 

#### 3.1.3. The Role of Chromogranin-A

Moreover, circulating levels of chromogranin-A (CgA) were usually increased by over-activating the SNS induced by stress stimulation under certain physiological and psychological conditions [64]. Nevertheless, it was found that the admission level of CgA, which is a neurohumoral marker, was lower in the TTS group compared to patients with anterior ST-elevation myocardial infarction (STEMI), and the CgA level in TTS patients was positively correlated with NT-Pro-BNP, indicating that CgA is co-secreted with the B natriuretic peptide (BNP), and CgA may be a local factor expressed by cardiomyocytes, which interact with intracardiac catecholamines [65,66,67,68]. Furthermore, an increased local release of catecholamines from the heart of patients with TTS-like LV dysfunction could lead to transient LV apical ballooning by mediating myocardial injury, which may be another potential mechanism of TTS [46,47,69,70,71]. Therefore, a greater myocardial, rather than adrenal catecholamine, release may be another potential mechanism for the occurrence of TTS. Of note, further basic and clinical studies are needed to clarify the effect of CgA on myocardial recovery during TTS.

#### 3.1.4. The Role of Alpha- or Beta-Adrenoceptors

The activation of alpha- or beta-adrenoceptors (α/β AR) is the primary trigger of emotional stress-induced cardiac changes. Under physiological conditions, catecholamines can enhance myocardial contractions by acting on the myocardial β1/β2 receptor, but excessive catecholamines can cause weakened ventricular wall motion at the apex and increased basal motion. Importantly, the physiological basis for this abnormal contraction was discussed, as the apical β2 receptor/β1 receptor density is higher than that of the base, and the sensitivity of the apical adrenergic receptor to catecholamines is also significantly higher than that of the base [16]. A high adrenaline concentration triggered β2AR to switch its coupling from the Gs protein to inhibitory Gi protein [72,73,74]. Catecholamines acting on β-ARs displayed differential capacities towards pathway activation, referred to as stimulus trafficking (ligand-directed receptor trafficking), biased agonism (biased signaling), functional selectivity or ligand-directed signaling [75,76,77,78]. Although the negative inotropic effect produced by Gi inhibiting the production of Gs-cAMP is harmful from a mechanical point of view, the Gs-to-Gi switch may have antiapoptotic and antiarrhythmic effects, which may be a cardioprotective mechanism against β1AR-catecholamine cardiotoxicity [16,79,80,81,82]. Both the p38 MAPK and phosphatidylinositol 3-kinase (PI3K)/Akt pathways were related to the β2AR-Gi-mediated antiapoptotic effect of adult cardiomyocytes, and increased PI3K/Akt activation was shown in myocardial biopsy samples in acute TTS patients [83,84,85]. The selective blockade of β1 alleviated the extent of akinesia in a TTS rat model, whereas the inhibition of β2 adrenergic receptors did not, indicating that β1 adrenoceptors play a crucial role in the pathophysiology of TTS [86].

#### 3.1.5. The Role of Beta-Blockers

Surprisingly, some research reported that the application of β-blockers was rather harmful in TTS or not effective on either mortality or the recurrence rate or QTc interval changes [16,87,88,89,90]. Some articles mentioned that beta-blockers should be helpful [6,91], as they were effective against arrhythmia [90]. Therefore, whether β-blockers play a role in the management of patients with TTS remains controversial. Instead of β-blockers, ACE inhibitors/angiotensin receptor blockers (ACEi/ARB) may reduce the risk of recurrence [92], whereas Natale et al. reported that a combination of ACEi/ARB and beta-blockers (BBs) may reduce the recurrence rate of TTS [93,94]. Furthermore, a high concentration of catecholamine can prolong the action potential duration (APD), trigger arrhythmias and increase ROS production through activating alpha 1-adrenoceptors, suggesting that alpha 1-adrenoceptor signaling may play a critical role for arrhythmogenesis in the setting of TTS [95]. Our group also found that a high concentration of isoprenaline prolonged APD via increasing ROS production, enhancing the late sodium channel current and suppressing the transient outward potassium current (Ito) [14]. These results suggest that ROS participates in beta and alpha 1-adrenoceptor signaling at high concentrations of catecholamines, which may contribute to the pathogenesis of TTS. Incomplete recovery and the frequent recurrence of TTS patients are two important issues that affect the long-term course of TTS patients, which may be related to persistent inflammation and myocardial energy damage [94,96]. Therefore, further investigations such as inflammatory or energetic–metabolic pathways are needed to explore the appropriate therapy for TTS.

The clinical features of TTS were reproducible by the intravenous administration of catecholamines and beta-adrenergic agonists [16]. Additionally, catecholamines induced the apoptosis of vascular endothelial cells via the catecholamine/β-receptor/caspase-3 and catecholamine/Bcl-2/caspase cascades [97,98], showing endothelial dysfunction. The catecholamine-mediated mechanisms remain unclear. Further research needs to be carried out to study the mechanism by which adrenergic receptors exert beneficial or unfavorable effects.

### 3.2. Coronary Artery Spasm

The reason for coronary artery spasm phenomenon is multifactorial, such as the abnormality in the autonomic nervous system, endothelial dysfunction, inflammation, oxidative stress, smooth muscle hyperreactivity, atherosclerosis, thrombosis and genetic predisposition. Therefore, coronary artery and coronary microvascular spasm may also be one of the important pathogenesis of TTS. In clinical practice, a different quantity of acetylcholine was selectively injected into the left and right coronary artery, respectively, to provoke a spasm, which is helpful to assess the microvascular spasm or dysfunction as a diagnostic tool [99,100,101]. In a cohort of 48 TTS patients, the incidence of CAS was approximately 20% [102]. A failure of coronary vasomotor function was demonstrated in most TTS patients. TTS and anomalous coronary artery coexisted or recurred alternately in the same individual [101,103]. Whether TTS and coronary artery spasm are two expressions of the same disease or, rather, two separate entities with overlapping mechanisms is also unclear [104]. 

### 3.3. Coronary Microvascular Dysfunction

There are very few studies attempting to investigate the existence and characteristics of coronary microvascular responsiveness abnormalities in TTS patients [105,106]. Verna et al. evaluated the vascular reactivity of coronary arteries by combining the sequential application of acetylcholine (Ach), nitroglycerin and adenosine with angiography and intracoronary pressure-Doppler flow monitoring and by performing intravascular ultrasound (IVUS) imaging in 45 vessels, demonstrating that an abnormal microvascular function was seen in 83% patients [107]. The Thrombolysis In Myocardial Infarction (TIMI) frame count (TFC) has been used as a quantitative indicator to evaluate the microcirculatory function in TTS patients [108]. A microcirculatory analysis in patients with TTS was conducted by calculating the TFC in the left anterior descending coronary artery (LAD), circumflex artery, and right coronary artery. The results suggested that the corrected TIMI frame count (CTFC) was significantly higher in the LAD of patients with TTS, compared with the control individuals [108]. However, no difference was observed between the two groups in other vessels [108]. TFC might be as an important diagnostic marker to evaluate endothelial or microvascular dysfunction in TTS [109]. However, not all cases of TTS present microvascular dysfunction. Regarding the question of whether microvascular dysfunction is the cause or the result of TTS, there is still no prospective research evidence to address it.

### 3.4. Endothelial Dysfunction

Endothelial dysfunction is a pathological state of the endothelium, which is characterized by the imbalance between vasoconstriction and vasodilator factors [110], and plays a particularly important role in TTS via impairing macro- or microvascular function. Endothelial dysfunction is an early manifestation of atherosclerosis. Decreased vascular and myocardial nitric oxide bioavailability in response to increased ROS production mainly caused endothelial dysfunction [111,112]. 

To date, several methods have been used to assess endothelial dysfunction in the setting of TTS, like flow-mediated dilation (FMD) of the brachial artery, change in coronary blood flow (CBF) in response to intracoronary Ach, and the peripheral arterial tonometry (PAT) score in response to the mental stress test in patients with TTS [13,106,113], which only occur in humans. Endothelin is mainly related to endothelial cell (EC) dysfunction, which can be prevented by selective endothelin-A receptor antagonism [114]. The elevation of endothelin-1 during the acute phase of TTS is similar to the level of AMI patients. The endothelin-1 level might only be increased acutely and normalized after the resolution of myocardial dysfunction. In addition, ROS also causes redox imbalance through several molecular mechanisms, such as the activation of proinflammatory signaling pathways and increased secretion of proinflammatory cytokines, which also exert harmful effects on EC [115]. This suggests that increased ROS production due to increased catecholamine release in the setting of TTS may injure myocardial cells and cause a transient impairment of myocardial contraction due to a transient coronary and peripheral endothelial dysfunction [116]. With respect to the regulation of vascular tone/endothelial dysfunction, it has been revealed that the level of miRNA125a-5p decreased, while the plasma level of its target endothelin-1 increased, which are in line with the microvascular spasm hypothesis [117]. Moreover, a significant increase in endothelial dysfunction was observed in TTS compared to the matched controls, while arterial stiffness, intima-media thickness, quality of life and laboratory markers are not significantly different [13]. In women with TTS, decreased citrulline production and increased thrombomodulin concentration are the characteristic of endothelial dysfunction, but there is no change in the prostacyclin levels, suggesting the presence of endothelial perturbation in TTS patients even in the long term [118]. It seems that, during menopause, decreased estrogen levels lead to increased sympathetic nerve activity and endothelial dysfunction. Thus, it is still necessary to focus on reversing endothelial dysfunction and/or on improving the efficacy of therapeutics such as ACE-inhibitors, AT1-receptor blockers or statins [116].

### 3.5. The Low Estradiol Level

Reduction of the estrogen level may be the main reason for the clinical phenomenon that TTS occurs frequently in middle-aged and elderly women, especially in women after perimenopause. A series of studies in ovariectomized (OVX) rats and ovariectomized rats plus estrogen application (OVX+E) were performed to explore the role of estradiol. The percentage contraction in the left ventriculography decreased significantly in the OVX group but not in the OVX+E group in response to pressure [119]. The elevated serum estradiol level can reduce cardiac changes caused by emotional stress [119]. However, Möller et al. reported that there was no difference between female TTS and AMI patients concerning the concentrations of E1 and E2. This suggested that altered sex hormone levels, especially an estradiol deficiency, could not be identified as a risk factor for TTS [120]. Importantly, most patients with electroconvulsive therapy (ECT)-induced TTS are women over 70 years of age receiving treatment for major depression [121,122,123]. That means emotion may be related to the estrogen level. Possibly, estrogen modulates cardiovascular reactivity through multiple pathways, such as increasing the vagal tone and decreasing the sympathetic nervous activity, as well as endothelial dysfunction. Therefore, more research on the mechanism by which estrogen affects TTS is needed.

### 3.6. Genetic Factors

Currently, a genetic predisposition has been suggested based on several familial TTS cases [124,125,126]. Furthermore, most postmenopausal women experience acute stressful events, but only a small percentage of them will develop TTS. Some women have premenopausal TTS episodes, which means that the intrinsic pathogenic mechanism does not only depend on the hormonal environment. Importantly, 11% of TTS patients relapse [127]. These phenomena strongly suggest genetically mediated vulnerability.

It has been proven that the rs17098707 polymorphism of the G protein-coupled receptor kinase 5 gene exists at a significantly higher frequency in TTS patients, providing preliminary evidence for a genetic influence in TTS [128]. A small number of families provide clinical evidence of a genetic predisposition toward TTS [124,125,129]. The polymorphisms are potentially involved in the pathogenesis of TTS via affecting adrenergic receptors located on cell membranes, which exist as several subtypes (α, β1, β2 and β3). After catecholamines bind to receptors, intracellular signals are mediated by adrenergic receptors β1 and β2 that couple with the Gα subunit of the Gs protein complex. At the same time, catecholamines induce the phosphorylation of G protein-coupled receptor kinase (GRK) to negatively regulate the signal, which shows a link between a polymorphism of one of the protein kinases mainly expressed in the heart and stress-induced acute ventricular dysfunction [128]. Moreover, the upregulation of transcriptional factors/immediate early genes, such as Fos, Jun, junB, Egr1 (NGFI-A), Nr4a1 (NGFI-B) and other genes, is involved in the adaptive and protective responses to the blood vessels and damage in the heart [130]. Eitel et al. reported for the first time preliminary (genome-wide association study) GWAS results of the largest genotyped TTS cohort, which is an important step forward to further elucidate a potential genetic predisposition for the development of TTS [131]. Borchert et al. identified highly relevant variants in cardiac genes, such as the RNA-binding motif protein 20 (RBM20) with a substitution of the highly conserved tyrosine at position 347 by a cytosine (p.Y347C) or calsequestrin 2 (CASQ2) having functional consequences for cytosolic Ca2^+^ and human ether-a-go-go-related channels (hERG) in TTS patients [132,133]. These will pave the way for ultimately assessing a potential genetic cause of TTS.

The finding of variants in genes encoding important cardiac proteins may underlie the hypothesis of a genetic predisposition toward TTS but also indicates a number of underlying mechanisms. Therefore, TTS is more likely to be a multifactorial disease caused by both genetic and environmental factors. Although the studies described here are promising for identifying genetic polymorphisms related to the development of TTS, the limitation is that the size of the subjects is small. Currently, we have some understanding of the genetic basis of TTS, but the process of its participation is very complicated. Whether genetics is really related to the pathogenesis of TTS and what roles it plays need to be further studied.

### 3.7. Other Possible Contributors

#### 3.7.1. Inflammation in TTS

It has been reported that TTS is characterized by a myocardial macrophage inflammatory infiltrate, changes in the distribution of monocyte subsets and an increase in systemic proinflammatory cytokines. Although, at 5 months, ultrasmall superparamagnetic particles of iron oxide (USPIO) enhancement were no longer detectable in the LV, persistent elevations in the serum interleukin-6 concentrations and reductions in the intermediate CD14^++^/CD16^+^ monocytes were detected, suggesting that TTS contains a chronic inflammatory process [15]. Moreover, data from a cardiovascular magnetic resonance imaging (CMR) presented inflammation in TTS patients [134], suggesting that systemic inflammation is a possible mechanism of TTS [135]. Subjects with TTS during follow-up have higher serum levels of IL-6 and IL-10 at admission [136]. Besides, the IL-7 levels in TTS patients were significantly elevated at 2–4 days after admission [137]. A higher concentration of IL-6 was related to the LV dysfunction, lowered left ventricular ejection fraction (LVEF) and cardiac functional class [138,139]. Santoro et al. evaluated the circulating levels of several inflammatory (IL-1β, IL-1α, IL-6, interferon(IFN)-γ, tumor necrosis factor (TNF)-α and vascular endothelial growth factor (VEGF)) and anti-inflammatory (IL-2, IL-4 and IL-10) cytokines; chemoattract component (EGF) and chemokines (IL-8 and MCP1) during the acute and subacute phases of TTS in comparison to ACS and found that the levels of IL-2 and IFN-γ were increased, while the IL-8, EGF IL-6, TNF-α and VEGF serum levels were reduced during the subacute phase compared to the acute phase [140]. Both localized and systemic inflammatory responses in the TTS were shown, presenting myocardial macrophages (mainly M1 macrophages) that were infiltrated, and the distribution of the monocytes was changed, and the systemic proinflammatory cytokines IL-6, IL-8 and CXCL1 (GROα) were increased, and some of these changes could persist for at least 5 months [15,78,141]. In addition, a significant association between autoimmune diseases and TTS was observed, which suggests also a link between inflammation and TTS [142].

#### 3.7.2. Abnormal Myocardial Metabolism in TTS

Both NO generation and effect were accentuated in TTS patients, which are related to β2-adrenergic receptor stimulation, as β2-adrenergic receptors are associated with the activation of nitric oxide synthase (NOS) [85,143,144]. In the presence of the superoxide (O_2_^−^) anion, catecholamines on the myocardium increased the release of NO-inducing formation of the peroxynitrite (ONOO^−^) anion, which is related to protein nitration, and deoxyribonucleic acid damage, resulting in poly (ADP-ribose) polymerase (PARP)-1 activation, which is a potential mechanism of energetic impairment [145,146,147,148,149,150,151]. These suggest that nitrosative stress may be critical to both inflammation and energetic impairment in TTS. Moreover, the activation of PARP-1 consumes adenosine triphosphate and inhibits glycolysis during the production of PAR [152,153,154]. Therefore, the inhibition of PARP-1 may ameliorate TTS and limit its recurrence. Nef et al. reported that sarcolipin (SLN), showing a remarkable ventricular expression in TTS patients, was increased in the acute phase compared with biopsies taken after the functional recovery of TTS and PLN phosphorylation was reduced, resulting in a reduced SERCA2a activity and its Ca(2+) affinity, but the interaction between SLN and PLN is not fully understood and needs to be clarified [155]. They also identified that catecholamine-mediated microcirculatory disturbance followed by ischemia and the direct toxicity of catecholamines may render the myocardium from the patients in the acute stage of TTS to severe morphological alterations, such as the disorganization of contractile and cytoskeletal proteins, and the affected myocardium showed a nearly complete reversibility [156]. To gain insight into TTS pathophysiology using ^1^H NMR-based metabolomics, Nuñez-Gil et al. demonstrated that the elevated acetate in the acute phase of TTS patients led to reduced energy production and subsequent myocardial stunning, while AMI patients showed elevated lipids, suggesting that the metabolic phenotype of TTS is different from acute myocardial infarction [157]. Matsuo et al. observed impaired metabolism in the apical region by (123)I-beta-methy-iodophenyl pentadecanoic acid ((123)I-BMIPP) imaging, which may result from a myocardial metabolism disorder caused by a surge of catecholamines or microvasospasm [158]. Furthermore, N-terminal pro-B-type natriuretic peptide/B-type natriuretic peptide (NT-pro-BNP/BNP) increased significantly during the first 24 hours after the onset of TTS, which is related to the level of increased catecholamines and the severity of LV contractile dysfunction [159].

#### 3.7.3. Cancer

Cancer and TTS are considered to also be closely related based on some studies [34,160,161,162,163,164]. The prevalence of neoplasms in TTS patients seems to be high [165]. Cancer can be found in up to 18% of TTS patients [166]. The diagnosis of cancer at the TTS event or during follow-up was predictive for a higher rate of the composite endpoint, indicating that the prevalence of cancer is high in TTS patients and is a risk factor for worse outcomes [35]. In cancer patients, the emotional stress of receiving a cancer diagnosis, paraneoplastic syndrome, pain syndromes, surgical treatment or chemotherapy may lead to TTS [167,168,169,170,171]. A neoplastic marker, carbohydrate antigen (CA)-125, may be useful for the early risk stratification of TTS subjects, because CA-125 may reflect the inflammatory state, prediction of the hospital stay duration, incidence of adverse events at follow-up and general clinical conditions in patients with TTS [172]. A group of nerve structures in the limbic system govern the response to stressors (such as those that may trigger TTS), especially the amygdala [53]. Radfar et al. employed 18F-FDG-PET/CT imaging and found that the higher amygdalar activity (AmygA) was related to an increased risk of TTS among the people with a high incidence of malignancy [142]. In addition to CA-125, Santoro and coworkers found that higher carcinoembryonic antigen (CEA) and carbohydrate antigen (CA)-19.9 levels were related to an increased risk of death at long-term follow-up in TTS patients [173]. Chemotherapy and radiotherapy could also cause coronary artery endothelial dysfunction that can manifest as TTS [167,174,175]. Furthermore, an overdose of cytokines, free radicals, prostaglandins, catecholamines and growth factors related to chemotherapy could exacerbate the deterioration of adrenergic receptor sensitivity and lead to the clinical manifestations of TTS [176,177,178,179,180,181]. Therefore, cancer screening may be useful for those patients who do not have a clear TTS-triggering stressor. However, some questions about the correlation between TTS and cancer remain unclear. For example, it is not clear whether the cause of death of TTS with a cancer is a cardiovascular or a non-cardiovascular cause of death, and the underlying mechanisms require further clarification.

Undeniably, a single mechanism cannot fully explain the pathogenesis of TTS, which should be the result of multiple factors. Although the pathological changes of TTS are reversible and the prognosis is relatively good, serious complications such as cardiogenic shock and sudden death are still observed in clinical practice. Importantly, there is no definite and effective prevention and treatment strategy. Thus, much remains to be discovered about its pathophysiological mechanism.

## 4. Experimental Models of TTS

Clarifying the pathophysiological mechanism of TTS is helpful for early diagnosis and therapeutic intervention. Preclinical studies using various in vivo or in vitro models are needed to understand the underlying pathophysiology of TTS and to search for drug treatment as a concept of precision medicine. Currently, the preclinical models related to TTS mainly focus on animal models, hiPSC-CMs models, other cell models and computational models, which are useful to probe and decipher the aspects of human disease. 

### 4.1. Animal Models and Mechanistic Studies

Experimental animal models may be a valuable tool for studying human diseases, which can be potentially used to clarify one or more specific questions. Compared with human models, animal models are relatively easier to manage and highly available. In addition, many animals are suitable for studying human diseases due to their high similarity in terms of anatomical basis and physiological functions with humans. Thus, the use of animal models can undoubtedly provide new insights for studying and elucidating the important pathophysiological mechanisms of TTS. 

In recent years, some researchers have already used animal models treated with high doses of catecholamines or adrenoceptor agonists or immobilization (IMO) to stimulate some or all of the abovementioned clinical manifestations of TTS. IMO in rats was used to study psychological stress, which is a successful model of TTS in rats [152,153,154,182,183,184,185,186,187]. IMO in rats triggered a transient and reversible reduction of LV contraction, including LV apical ballooning prevented by pretreatment with an adrenoceptor blocker, suggesting that the activation of β1 adrenergic receptors in the heart and the activation of α1 adrenergic receptors in the aorta are the primary cause of TTS [183,188,189]. 

Furthermore, ovariectomized (OVX) and estradiol-supplemented ovariectomized female rats (OVX+E) with IMO stress were used as an animal model of TTS, finding that the percentage contraction in the left ventriculography of OVX rats was significantly reduced in response to stress, while the mRNA expression of c-fos, a marker of cellular activation, in the OVX+E rats was significantly decreased in the paraventricular hypothalamic nucleus, adrenal gland and LV, suggesting that an increase of estrogen can attenuate the emotional stress-induced hypothalamo–sympatho–adrenal outflow from the central nervous system to the target organs [154]. 

Of note, the isoprenaline (ISO)-induced female TTS rat model needs a higher triggering dose and has a lower mortality than that of male TTS rats [190]. This may be related to the fact that estrogen has a vasodilator effect through the induction and activation of endothelial nitric oxide synthase, and the reduction of estrogen may interfere with coronary microcirculation by indirect action on the nervous system and by direct action on the heart vessels [119,187].

Another possible mechanism is that estrogen protects the myocardium from TTS by increasing the activity of the β2 AR-Gas signaling pathway and reducing the concentration of catecholamines in plasma [191]. Repeated intravenous infusion with epinephrine in cynomolgus monkeys induced LV dysfunction with apical ballooning and wall motion abnormalities, which mimics that of TTS in clinical cases [192]. This model is of great value in understanding the pathogenesis of TTS associated with sympathomimetic hyperfunction in non-human primate models. In addition, a worthy and novel experimental model for inverted TTS-like cardiomyopathy is the ventricular tachyarrhythmia-related inverted cardiomyopathy in rabbits with vagal stimulation. Cardiac lesions related to ventricular tachyarrhythmias were involved in the basal portion, mitral valve and papillary muscles but not the apex [193]. Nevertheless, an α-adrenergic blockade reduced the development of the adrenaline-induced cardiac basal lesion but did not affect the structural and functional alterations [194]. Importantly, epinephrine-specific β2AR-G(i) signaling may have evolved as a cardioprotective strategy to limit catecholamine-induced myocardial toxicity during acute stress, while α2AR/Gi-dependent signaling attenuates myosin-binding protein-C (MyBP-C) phosphorylation and contractility in the anterior wall (AW) through an epinephrine surge in TTS rats [16,182]. However, no statistically significant difference of the β2AR-dependent cAMP levels was observed between the apical and basal cells [191].

Moreover, a single injection of isoproterenol in mice could induce TTS-like regional dyskinesia, and the study demonstrated that lipotoxicity is closely related to catecholamine-induced myocardial dysfunction, including neurogenic stunning, metabolic stunning and electrophysiological stunning [195]. The direct inhibition of myocardial ApoB lipoproteins and subsequent reduction in lipid export by the supraphysiological level of catecholamines may result in cardiac lipotoxicity.

Emotional stress and a surge of catecholamine upregulated oxidative stress-related factor-heme oxygenase-1 (HO-1) in the heart, while the blockade of α- and β-adrenoceptors attenuated this effect [152], suggest that oxidative stress plays an important role in the occurrence and development of TTS. Recent evidence indicated that TTS patients suffered from acute endogenous catecholamine release, which may trigger oxidative stress and inflammation. The apoptosis of inflammatory cells was visible in the whole course of TTS, while the apoptosis of endothelial cells appeared only in the recovery phase [196]. ISO significantly increased the levels of reactive oxygen species (ROS) in the setting of TTS, leading to the injury of myocardial cells, mitochondrial dysfunction, acute Ca^2+^ overload, TLR4/NF-κB signaling alterations, dysregulation of the glucose and lipid metabolic pathways represented by decreases in the final glycolytic and β-oxidation metabolites and reduced availability of Krebs cycle intermediates. It was shown that icariin, an antioxidant and anti-inflammatory agent, prevented ISO-induced TTS-like cardiac dysfunction in rats by suppressing the TLR4/NF-κB pathway and long-term inhibition of PI3K/AKT/mTOR expression and by reducing the mitochondrial ROS and oxidative stress-induced apoptosis, which provides new insights into the protective effect against myocardial dysfunction in TTS rats [197,198,199,200,201]. 

Early treatment with isoflurane reduced left ventricular dyskinesia and improved the survival rate of experimental TTS, while H_2_S reduced ROS formation by reducing NADPH oxidase [202,203]. GPER, azelnidipine, Tempol and amlodipine also played a protective role for TTS [153,186,204,205]. In terms of mechanism, this effect is mediated by balancing the coupling of β2AR with the Gαs and Gαi signaling pathways [204].

In the TTS animal models, the subjects must be killed for testing certain biomarkers. Interestingly, speckle tracking echocardiography (STE) and global longitudinal strain (GLS) can be used to quantitatively detect subtle myocardial abnormalities and provide a reliable, noninvasive method to predict early myocardial injury in stress cardiomyopathy (SCM) rats, showing that ISO can reduce GLS and circumferential (GCS) strains of males and females [185,206]. 

The animal models of TTS are mainly based on monkeys, rabbits, rats and mice, which can be used to simulate TTS to a certain extent through immobilization, repeated intravenous infusion of an epinephrine overdose, vagal stimulation, intraperitoneally isoprenaline and other methods [152,153,154,182,183,184,185,186,187,188,190,191,192,193,194,195,197,198,199,200,201,202,203,204,205,206]. Many live-stranded cetaceans and terrestrial wildlife experienced capture myopathy, which was similar to TTS [207,208]. Nevertheless, the animal models of TTS cannot fully replicate the characteristics of human TTS and clarify the mysterious etiology of its human manifestations. There is a huge species difference between them and humans. In addition, different aspects were not explored in these animal models, including the corresponding mild-to-moderate release of cardiac biomarkers, the natural course of the electrocardiographic manifestations, the complete recovery of LV function and the presence and the degree of myocardial edema, all of which have been observed in patients with TTS. Therefore, other models are essential, although these animal models can simulate TTS to a certain extent and provide the basis for pathophysiological studies.

### 4.2. Human Cardiomyocytes Derived from Induced Pluripotent Stem Cells (hiPSC-CMs) Models

The hiPSC-CM possess a lot of advantages in modeling genetic cardiac disorders like long-QT syndrome, short QT syndrome, arrhythmogenic right ventricular cardiomyopathy (ARVC), Brugada syndrome and familial hypertrophic cardiomyopathy (HCM) [209,210,211,212,213,214,215,216,217,218,219]. First, hiPSC-CMs possess a human gene background, avoiding possible effects resulting from the gene differences between humans and animals. Second, hiPSC-CMs provide a better tool for cardiac drug screening and ion channel research compared with Xenopus oocytes, human embryonic kidney (HEK) cells and Chinese hamster ovary (CHO) cells, which lack cardiac ion channel macromolecular complexes. Third, disease-specific or person-specific hiPSC-CMs can be generated, and patient-specific mechanistic study and drug testing can be performed, suggesting their significance for precision medicine. Additionally, genome editing combined with patient-specific iPSC-CMs has allowed exploring the genotype–phenotype correlation of unknown gene mutations or variants in patients. 

So far, only several TTS-relating studies using hiPSC-CMs have been reported [25,95,204]. In the last few years, TTS was not regarded as a genetic disease. However, increasing evidence suggests the involvement of genetic factors in TTS. For example, highly relevant variants, such as *RBM20*, encoding RNA-binding motif protein 20, or *CASQ2*, encoding calsequestrin 2, exist in TTS patients [132]. Furthermore, acquired long QT syndrome has been described in up to 70% of TTS cases and is associated with life-threatening arrhythmias (LTA) triggered by concomitant atrial fibrillation. El-Battrawy et al. used hiPSC-CMs to investigate the toxic effects of catecholamine on cellular electrophysiological properties and the protective effects of estrogen, finding that estrogen may reduce the sensitivity of cardiomyocytes to catecholamine by reducing adrenoceptor expression [14]. The pathogenesis process of arrhythmias was induced by catecholamine excess, which caused the overstimulation of adrenoceptors, increase in ROS production, ion channel dysfunction (enhancement of late I_Na_), APD/QT prolongation and tachyarrhythmias. Their study suggests that ROS-blocker, β-blocker and late sodium channel blocker may be potential alternatives to treat arrhythmias induced by catecholamine excess. 

Another TTS model in hiPSC-CMs was developed with high doses of epinephrine, identifying that G1/estrogen (E2) alleviated epinephrine-induced cardiac damage via reducing the brain natriuretic peptide in plasma and released lactate dehydrogenase into the culture supernatant [204]. Huang et al. used a toxic concentration of epinephrine to mimic the setting of TTS, finding that high concentrations of epinephrine prolonged APD and induced arrhythmia events [95]. The effect of epinephrine was attenuated by alpha-adrenergic receptor blockers phentolamine [95]. An activator of alpha-1, but not alpha-2, receptor mimicked epinephrine effects, indicating that the alpha-1 receptor contributes to arrhythmogenesis of TTS. Further, it was revealed that NADPH-ROS-PKC signaling mediated the effects of the alpha-1 receptor [95,220], which may provide new insights for the treatment of TTS.

**Figure 2:**
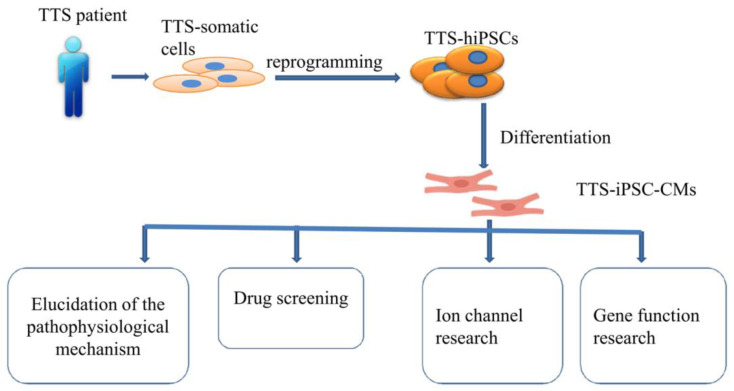
Overview of human-induced pluripotent stem cell-derived cardiomyocyte (hiPSC-CM) model.

Of note, whether hiPSC-CMs from TTS patients can recapitulate some features of the disease need to be investigated. Borchert et al. used, for the first time, hiPSC-CMs generated from two TTS patients for the study, revealing that β-adrenergic signaling, including the cAMP response and cAMP-dependent PKA activity, was increased in TTS-iPSC-CMs after treatment with high levels of catecholamines [132]. The enhanced β-adrenergic signaling in TTS-iPSC-CMs under catecholamine-induced stress increased the cardiac stress marker NR4A1 expression [132]. These data strongly support applications of hiPSC-CMs for TTS studies and also suggests the possible involvement of genetic factors for the pathogenesis of TTS. Catecholamine-treated hiPSC-CMs or TTS-specific iPSC-CMs mimicked features consistent with those found in individuals with TTS. Additionally, TTS-iPSC-CMs provide a promising and reliable cell source for elucidation of the pathophysiological mechanism, drug screening, ion channel research and gene function research (Figure 2).

However, hiPSC-CMs have several limitations, such as a lack of t-tubular network, polygonal shapes and rhythmic automaticity. Importantly, to overcome the limitations of a relatively immature fetal-like phenotype of hiPSC-CMs, extending the cell culture time [221], using a medium containing galactose and fatty acids [222], electrical field stimulation [223], electric pacing and mechanical stimulation [224], extracellular matrix [225] and 3D cardiac tissue with electric stimulation [226] have been used to improve the maturity of hiPSC-CMs.

HiPSC-CMs can overcome the limitations of artificial animal or other in vitro TTS models. Therefore, hiPSC-CMs provide a very helpful and easily available disease research model for translational medicine, which can bridge the gap between basic research and clinical disease.

### 4.3. Other Models

In addition to hiPSC-CMs, H9C2 cells can also be used to study the pathophysiological mechanism of TTS. Pretreatment with Tempol can reduce the production of reactive oxygen species and the deposition of lipid droplets and protect the mitochondrial function by reducing mitochondrial swelling, which provides a theoretical basis for Tempol to prevent isoproterenol-induced TTS [205]. Additionally, a computational model of the rat LV in TTS was used to investigate the mechanisms of the typical shape of the ventricle observed in TTS, finding that three potential dominant mechanisms are related to the effects of β-adrenergic stimulation, for example, the apical–basal variation of calcium transients, apical–basal variation of calcium sensitivity and apical–basal variation in maximal active tension [227]. The data from computational models can be used to interpret future experimental data and provide a theoretical basis for basic experiments.

At present, from the study of TTS models, scientists have demonstrated that some known mechanisms related to the pathogenesis of TTS from those models include a surge in catecholamines, endothelial dysfunction, estradiol levels, coronary microvascular dysfunction, apoptosis in CMs, etc. However, the animal or cell models of TTS that can mimic the characteristics of clinical TTS are limited. Importantly, researchers have made a certain level of discoveries from animal models to cellular models, which provides strong evidence for elucidating the pathophysiological mechanism of TTS (Table 1).

### 4.4. Conclusions and Future Perspectives

Taken together, TTS is a recently discovered type of heart disease that mimics acute myocardial infarction, but no relevant lesions were observed in the coronary arteries. Many studies have been conducted to explore its underlying mechanism, but the exact pathophysiological mechanism of TTS remains controversial. However, the existing studies have shown that its underlying mechanisms include sympathetic hyperexcitability, direct myocardial toxicity caused by catecholamine, coronary microvasospasm and dysfunction, estrogen deficiency, endothelial dysfunction and genetic factors. Recently, cancers and COVID-19 have been found to be related to the onset of TTS. TTS seems to be the result of a combination of multiple mechanisms, which makes the treatment of this disease nonspecific.

The acute phase of TTS can cause death due to cardiogenic shock, ventricular fibrillation, acute pulmonary edema and cardiac arrest and other noncardiac causes. Acute TTS is also related to increased levels of C-reactive protein and the leukocyte count, as well as increased norepinephrine, suggesting that catecholamines might cause a more systemic inflammatory response. Furthermore, the reason why older women are more susceptible to TTS remains unresolved. It is necessary to further explore the cardiac endocrine pathways, which may provide important clues for genetic/environmental sensitivity. Thus, there is an urgent requirement for some suitable experimental models to simulate TTS in order to understand the pathogenesis of the disease and provide ideas for the treatment of the disease.

Currently, we noticed that the disease models we know about TTS are mainly animal models, such as rats, mice, monkeys and rabbits, and cell models like hiPSC-CMs. Undeniably, these models have their own advantages and limitations. Whether those models really replicate the human TTS entity or only reflect the toxic effects of catecholamines is still an open question. Similarly, hiPSC-CMs have several limitations in TTS research, such as an immature phenotype. However, it is undeniable that experimental models play an irreplaceable role in studying the pathophysiological mechanism of TTS, because translational research is useful for future precision medicine. Of note, preclinical models that help us clarify the exact mechanism may provide a unique and exciting opportunity for PM and play an important role in improving the diagnosis and treatment of TTS. Importantly, more efforts should be made to improve the existing models or find new models combining with new techniques for future studies in TTS.

## Figures and Tables

**Table 1:** Experimental models for TTS.

Model		Method	Main Finding
Animal model	Rats [153,182,183,186,187,188,189]	Immobilization (IMO)	(1) The activation of β1 adrenergic receptors in the heart and the activation of α1 adrenergic receptors in the aorta were the primary cause of TTS (2) The reduction of estrogen may interfere with coronary microcirculation and may be involved in the primary cause of TTS by indirect action on the nervous system and by direct action on the heart (3) α2AR/Gi-dependent signaling attenuated myosin-binding protein-C(MyBP-C) phosphorylation and contractility in the anterior wall (AW) through an epinephrine surge in TTS rats
	Ovariectomized (OVX) and estradiol-supplemented ovariectomized female rats [152,154]	Immobilization (IMO)	(1) The reduction of LV contractility and the increase of heart rate in response to emotional stress were attenuated by supplement of estradiol in the ovariectomized rats. (2) Emotional stress and a surge of catecholamine upregulated heme oxygenase-1 (HO-1)
	Cynomolgus monkeys [192]	Intravenous infusion of epinephrine overdose	LV dysfunction with apical ballooning and wall motion abnormalities
	Rabbits [193]	Vagal stimulation	The cardiac lesions related to ventricular arrhythmias were involved in the basal portion, mitral valve, and papillary muscles but not the apex
	Mice [185,195,206]	A single dose injection of isoprenaline	(1) Lipotoxicity was closely related to catecholamine-induced myocardial dysfunction, including neurogenic stunning, metabolic stunning, and electrophysiological stunning (2) ISO reduced GLS and circumferential (GCS) strains of males and females
	Rats [190,194,197,198,199,200,201,202,203,205]	Isoprenaline (ISO)	(1) TTS rats had significantly lower left ventricular end-diastolic pressure and significantly better estimates of cardiac function. (2) Its apical perfusion was not impaired in the early stage of TTS (3) ISO significantly increased the levels of reactive oxygen species (ROS) in the setting of TTS (4) Early treatment with isoflurane could reduce LV dyskinesia and improve the survival rate of experimental TTS (5) GPER, azelnidipine, Tempol and amlodipine also played a protective role for TTS
	Rats [204]	Epinephrine	GPER played a protective role against TTS
hiPSC-CMs models	hiPSC-CMs models [14]	Isoprenaline	Estradiol had protective effects against catecholamine excess and hence reduction in estrogen level may increase the risk of acquired long QT syndrome in TTC
	hiPSC-CMs models [204]	Epinephrine	Knockdown of GPER by siRNA abolished E2 effects on increasing ICa-L and action potential duration in the stress state
	hiPSC-CMs models [95]	Epinephrine	High concentrations of epinephrine inhibited the depolarization rate in hiPSC-CMs, the duration of action potentials and induced arrhythmia events while the effect of epinephrine was attenuated by alpha-adrenergic receptor blockers-phentolamine
	TTS-iPSC-CMs [132]		The β-adrenergic signaling, including cAMP response and cAMP-dependent PKA activity, was increased in TTS-iPSC-CMs
Other cells model	H9C2 [205]	Isoproterenol	Pretreatment with Tempolcould reduce the production of reactive oxygen species and the deposition of lipid droplets and protect mitochondrial function by reducing mitochondrial swelling
	Computational model [227]		Three potential dominant mechanisms are related to the effects of β-adrenergic stimulation

## Data Availability

Not applicable.
